# Myelin Repair as a Neuroprotective Strategy for Multiple Sclerosis: From Bench to Bedside

**DOI:** 10.3390/medicina62061183

**Published:** 2026-06-18

**Authors:** Tima Battah, Vasilios Mastorodemos, Erich Struecker, Dimos Dimitrios Mitsikostas, Dimitrios Papadopoulos

**Affiliations:** 1 Medical School, European University Cyprus, Nicosia 2404, Cyprus; tb212403@students.euc.ac.cy (T.B.); es221686@students.euc.ac.cy (E.S.); 2 Mesogeios Diagnostic Center, 71306 Heraklion, Crete, Greece; vasmast@yahoo.com; 3 1st Neurology Department, Eginition University Hospital, National and Kapodistrian University of Athens, 15784 Athens, Attica, Greece; dmitsikostas@med.uoa.gr

**Keywords:** multiple sclerosis, demyelination, remyelination, neurodegeneration, neuroprotection, aging, cellular senescence

## Abstract

Multiple sclerosis (MS) is a neuro-inflammatory disease characterized by demyelination in the central nervous system (CNS). Although a substantial endogenous capacity for remyelination has been demonstrated, this process is frequently incomplete and exhibits marked intra- and inter-individual heterogeneity. Several factors influence the extent of spontaneous myelin regeneration, including age, sex, disease course, and lesion localization. Oligodendrocytes (OL), derived from oligodendrocyte progenitor cells (OPCs), are the principal myelinating cells of the CNS. The regenerative cascade involves several key stages, including OPC activation, recruitment, differentiation into oligodendrocytes (OL), and myelin deposition. This process is orchestrated in a spatiotemporal manner by a complex interplay of intracellular signaling pathways, genetic determinants, and dynamic microenvironmental cues, which together balance inhibitory and pro-remyelinating influences. Several lines of evidence indicate that chronically demyelinated axons are vulnerable to degeneration, whereas successful remyelination may confer neuroprotection. These observations underscore remyelination as a promising neuroprotective therapeutic target for preventing or slowing disability progression in MS, a condition in which gradual neuroaxonal degeneration is believed to underlie irreversible disability progression. In this review, we aim to bridge the gap between fundamental biological mechanisms of remyelination and their clinical relevance. We examine recent advances in in vivo techniques for assessing remyelination and discuss how these measures correlate with clinical and disability outcomes. In addition, we review recent clinical trials of remyelination-promoting therapies and analyze the challenges that have limited their advancement beyond phase II. Overall, we seek to provide a comprehensive overview of the remyelination process from bench to bedside, highlighting both the obstacles and the therapeutic potential of remyelination strategies in MS.

## 1. Remyelination: An Introduction to Endogenous Myelin Repair

**Figure 1:**
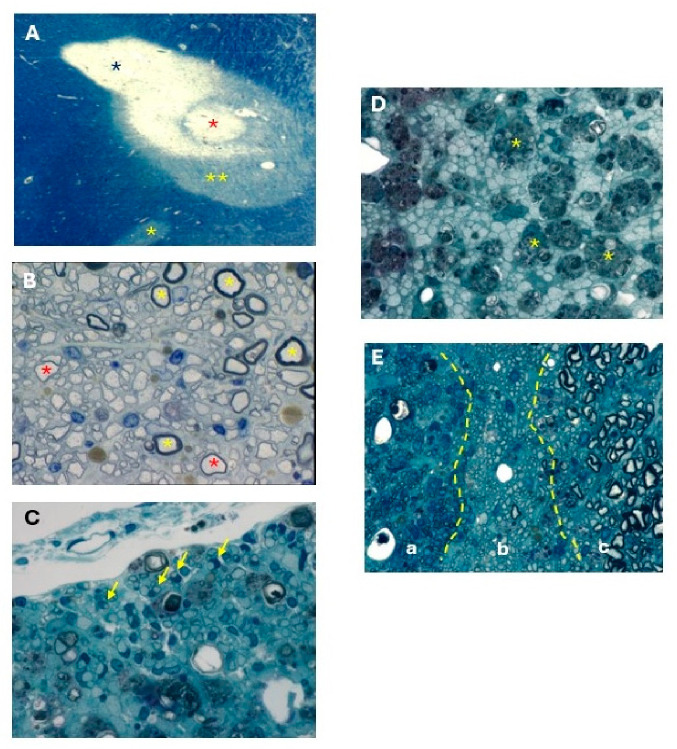
(**A**): Image of a section from a paraffin-embedded block from a chronic MS case, stained for myelin with Luxol fast blue (LFB) stain. White matter ovoid-shaped demyelinated lesion recognized by the lack of blue staining (black asterisk). Part of the periphery of the lesion exhibits pale blue staining characteristic of remyelination (yellow double asterisks). Part of the pale-remyelinated part of the lesion exhibits a lack of staining, probably indicating a second demyelinating attack on the remyelinated axons (red asterisk). Completely remyelinated lesion or shadow plaque (single yellow asterisk). (**B**): High-magnification image from a semithin toluidine blue-stained transverse section from the spinal cord of MOG-induced EAE in DA rats, showing a demyelinated lesion exhibiting oligodendrocyte-mediated remyelination recognized by their thinner myelin sheaths (red asterisks), compared to a few axons with apparently intact original myelin sheaths (yellow asterisks). (**C**): Transverse section of a demyelinated MOG-EAE lesion in the fasciculus gracillis showing signs of early Schwann cells-mediated remyelination indicated by thicker myelin sheath and Schwann cell nuclei and somata visible adjacent to remyelinated axons (yellow arrows) (**D**): High magnification image of a demyelinated lesion at an early stage of remyelination where all myelin debris is only visible within scavenging macrophages (examples of myelin debris-laden macrophages marked with yellow asterisks) (**E**): Fully remyelinated lesion in the dorsal funiculus of a MOG-EAE rat. Area remyelinated by Schwann cells indicated by thicker myelin sheaths and increased space between fibers, with Schwann cell nuclei and somata visible adjacent to remyelinated axons (a). Area of central type oligodendrocyte-mediated remyelination characterized by thinner-than-normal myelin sheaths and tight compaction of remyelinated axons (b). Part of the fasciculus cuneatus axons retaining their original thicker myelin sheaths (c). Areas (a), (b) and (c) separated by dashed lines. Image (**A**) from an MS autopsy tissue block provided by the UK Multiple Sclerosis tissue bank at Imperial College London, UK. Images (**B**–**E**) are Toluidine blue-stained, semithin transverse sections from the spinal cord of the MOG-EAE model in Dark Agouti rats from the collection of Dr. Papadopoulos.

Early observations of demyelinating pathologies revealed that demyelinated axons in both the peripheral and central nervous systems can undergo endogenous repair. This process, through which new myelin is generated around previously denuded axons, is known as remyelination [1,2]. In the central nervous system (CNS), remyelination has been described in demyelinating disorders such as multiple sclerosis (MS) and the leukodystrophies [3,4]. It is also a consistent feature of experimental models of demyelination, including immune-mediated models (e.g., experimental autoimmune encephalomyelitis, EAE), virally induced models (e.g., Theiler’s murine encephalomyelitis virus), toxin-mediated models (e.g., cuprizone ingestion or local injection of ethidium bromide or lysolecithin), and genetic models driven by myelin gene mutations in mice [5]. In 1961, functional recovery following experimentally induced partial paralysis in feline models was observed in parallel with the reappearance of myelin on previously demyelinated axons [5]. This pioneering ultrastructural study marked the beginning of modern myelin biology, demonstrating the regenerative capacity of the CNS in restoring the myelin sheath [5,6]. Shortly thereafter, Périer and Grégoire documented spontaneous remyelination in an ultrastructural analysis of MS plaques, providing critical confirmation of findings obtained from animal models [7]. Lesions that stain uniformly pale with Luxol Fast Blue were subsequently identified as remyelinated plaques, also referred to as shadow plaques (Figure 1A) [2].

Oligodendrocytes (OLs), the most abundant glial cells in the adult human brain, are responsible for the formation of the myelin sheath in the CNS. They are also found to be implicated in the remyelination process [6]. First observed by Virchow in the 1850s [8], these glial cells have been shown to play a crucial role in myelination, immunomodulation, and metabolic support of neurons [9]. In fact, the myelin sheath is the extension of the highly stabilized and specialized plasma membrane processes of OLs, winding around axons [9]. During development, OLs arise from oligodendrocyte progenitor cells (OPCs), which migrate prenatally and postnatally, in separate waves, to populate the telencephalon and the spinal cord [10]. OPCs remain in the adult brain, particularly in the subventricular zone (SVZ), and constitute up to 6–8% of the adult brain cell population [11,12].

Following demyelinating injury, lesioned areas show an increase in the number of proliferating OPCs [13]. As for OLs, evidence showed that despite their capacity to divide, pre-existing OLs at the site of injury do not proliferate, and distal OLs do not migrate to the affected area [14,15]. In fact, existing OLs surviving demyelinating injury in EAE mice showed limited remyelination potential, with an 8% gain in original myelin length and insufficient sporadic formation of myelin internodes [16]. Similarly, when transplanted to the site of injury in animal models, no myelin sheath was produced around demyelinated axons, and no evidence of cell migration was observed [17]. Nevertheless, recent studies have challenged this idea [18,19], showing that surviving mature OLs can extend processes and contribute to the remyelination of axons deprived of their myelin sheath [20].

Although the contribution of pre-existing OLs to remyelination remains ambiguous, the importance of generating new OLs after demyelinating injury is undeniable, as impaired formation of these cells from OPC differentiation is a key factor in pathologies such as multiple sclerosis. In areas experimentally deprived of OLs, remyelination is still possible, unless OPCs’ recruitment and differentiation are impaired [21]. OPC injection into demyelinated lesions in rats incapable of self-remyelination led to OL formation, which eventually remyelinated the affected axons [22]. The process is aided by glial cells (microglia, astrocytes) and inflammatory cells that interact with OPCs via chemokine signaling, the secreted extracellular matrix (ECM), and phagocytic activity, thereby promoting or inhibiting OPC maturation [23]. In fact, depletion of astrocytes or microglia delayed and prevented the repair process in demyelinated injury [24,25]. Similarly, astrocyte depletion prompts OPCs to differentiate into Schwann cells rather than OLs. Fundamentally, Schwann cells are the main myelinating agents of the peripheral nervous system (PNS) [26]. Nevertheless, Schwann cell-mediated CNS remyelination has been described in MS and is largely promoted by bone morphogenetic protein (BMP) signaling [27]. Schwann cell-mediated remyelination is highly efficient at restoring axonal function and at resisting recurrent demyelinating injuries, making it more advantageous than OL-mediated remyelination [27]. However, the process remains rare and is typically confined to destructive spinal cord lesions (Figure 1C,E), where Schwann cells may migrate to the injury site [28,29,30]. Overall, the discovery of the endogenous repair mechanism and its key players prompted further research into the underlying cellular and molecular pathways to enhance this process and target it therapeutically.

## 2. From Oligodendrocyte Progenitors to Mature Myelinating Oligodendrocytes

**Figure 2:**
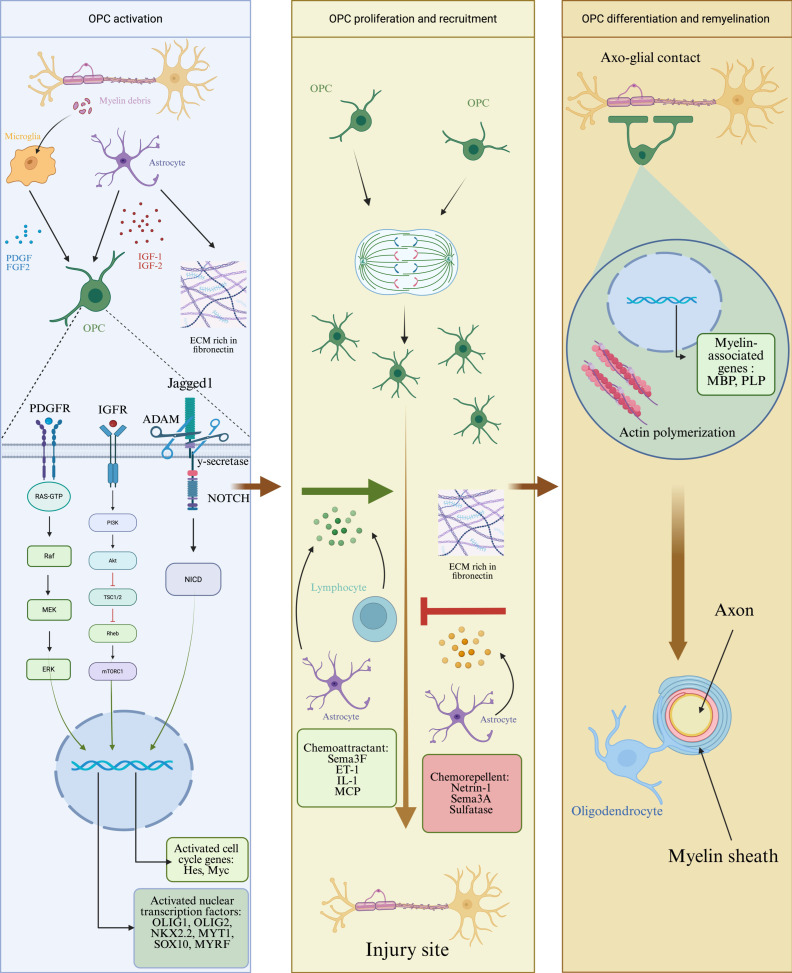
Multistep regulation of oligodendrocyte progenitor cells (OPC) differentiation and oligodendrocytes (OL)-mediated remyelination. Left column: OPC activation. Upon demyelinating injury, astrocytes and microglia are activated following phagocytosis of myelin debris. Reactive astrocytes and microglia secrete growth factors such as platelet-derived growth factor (PDGF), fibroblast growth factor (FGF2), insulin growth factor 1 (IGF-1) and IGF-2, as well as fibronectin-rich extracellular matrix (ECM). This triggers the activation of numerous intracellular signaling pathways, including the RAS-RAF-MEK-ERK, PI3KT-AKT-mTOR, and the Jagged1-NOTCH cascades. These are activating cascades promoting the initiation of OPC differentiation through the transcription of cell-cycle genes (e.g., HES, MYC) and oligodendroglial lineage factors (OLIG1/2, NKX2.2, MYT1, SOX10, MYRF). Middle column: OPC proliferation and recruitment. OPC proliferate following their activation and migrate to the site of injury under the influence of chemoattractants such as semaphorin 3F (sema3F), endothelin-1 (ET-1), interleukin-1(IL-1) and monocyte chemoattractant protein-1 (MCP-1). Similarly, chemorepellents secreted by astrocytes, lymphocytes or the ECM, such as netrin-1, semaphoring 3A (sema3A) and sulfatase oppose OPC migration. Right column: OPC differentiation and remyelination. Following axo-glial contact, myelin gene expression, such as myelin basic protein (MBP) and proteolipid protein (PLP), allows further cytoskeletal remodeling, leading to extension of OPC processes and axonal ensheathment.

The process of forming OLs by OPCs is divided into distinct steps: OPC activation, OPC recruitment, and OPC differentiation (Figure 2).

1: OPC activation: It is the switch of phenotype from a quiescent to a regenerative state characterized by increased mitogenic activity. This alteration is accompanied by changes in morphology, such as an increase in size and shortening of their processes [23]. As in developmental myelination, OPC activation is accompanied by increased expression of several key transcription factors, notably oligodendrocyte transcription factor 2 (OLIG2), NKX2.2, and myelin transcription factor 1 (MYT1) [5,12]. The alteration in OPCs’ phenotype is mediated by the combination of an inflammatory microenvironment and the activation of supporting cells, such as microglia and astrocytes [23,31]. Activated microglia phagocytose myelin debris, a process that removes the inhibitory impact that myelin debris exerts on the initiation of remyelination (Figure 1D) [23,31,32]. On the other hand, reactive astrocytes may secrete growth factors such as platelet-derived growth factor (PDGF) and fibroblast growth factor 2 (FGF2), further enhancing the recruitment and activation of microglia [5,12,23]. Activated astrocytes alter the composition of the ECM by secreting fibronectin, a promoter of OPC proliferation. Nevertheless, excessive deposition of fibronectin has been shown to impede myelin formation [33]. Additional cytokines released by microglia, such as insulin-like growth factor I and II (IGF I/II), transforming growth factor (TGF), and galectin, prevent OPCs’ apoptosis under the influence of the inflammatory microenvironment mediated by the lymphocytes at the site of injury and promote the expression of myelin protein and actin polymerization, respectively [23,34,35].

2: OPC recruitment: It is the process by which proliferating OPCs migrate to areas of demyelination and subsequently differentiate into OLs. OPCs migrate either from the SVZ or from the perilesional areas [23], with the latter being the dominant contributors to remyelinating OLs in widespread demyelinating disorders across the CNS [12]. Recruitment is governed by an integrated spatio-temporal signaling involving semaphorins, netrin-1, and sulfatase 2 [23]. Semaphorins, a group of guidance molecules secreted by astrocytes, particularly sema3A and sema3F, play a dual coordinating and antagonistic role as a recruiting and halting cue [23,36]. As for netrin-1 and sulfatase 2, they exert an inhibitory role both in the recruitment and the remyelination process [34,37]. During recruitment, negative guidance serves targeted lesion repair without depleting the precursors’ pool [23]. Additionally, cytokines secreted by the activated astrocytes, such as endothelin 1 (ET-1), interleukin (IL-1), monocyte chemoattractant protein (MCP), and C-C motif ligand (CCL2), favor OPC migration to the injury site [34].

**Figure 3:**
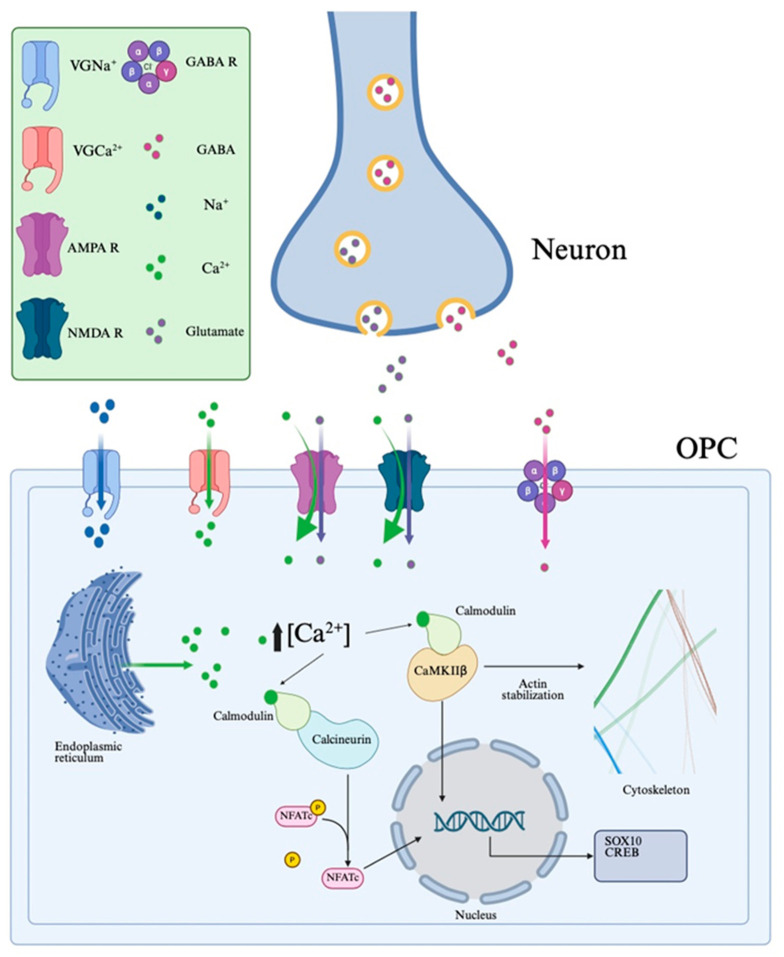
Axo-glial synaptic communication. Schematic modified from [35]. Oligodendrocyte progenitor cells (OPC) respond to an array of neurotransmitters secreted by neurons, such as glutamate and γ-aminobutyric acid (GABA). These act on OPC cell surface receptors, such as GABA-R, isoxazolepropionic acid receptor (AMPA-R) and N-methyl-D-aspartate receptors (NMDA), causing an influx of intracellular calcium Ca^2+^. Intracellular accumulation of Ca^2+^ and Na^+^ is further mediated by influx through voltage-gated Ca^2+^ (VGCa^2+^) and Na^+^ (VGNa^+^) channels, respectively, and an endoplasmic reticulum release of Ca^2+^. Increased Ca^2+^ binds calmodulin, which activates downstream calcineurin and calcium-calmodulin-dependent protein kinase II β (CaMKIIβ). Calcineurin dephosphorylates nuclear factor of activated T cells (NFATc), which otherwise inhibits transcription of SOX10. CaMKIIβ translocates to the nucleus and activates the CREB transcription factor. Moreover, CaMKIIβ binds actin filaments, enhancing their crosslinking and further stabilizing the cytoskeleton.

3: OPC differentiation: As for OPC differentiation, the process takes place through distinct steps: establishing a connection between an OPC and an axon, altering signaling pathways upstream of myelin-associated genes, and ultimately, myelin formation and ensheathment [23]. A multifaceted communication occurs between the neuronal axon and the OPC to establish the axo-glial contact, which includes both synaptic and non-synaptic interactions. In fact, OPCs are unique among glial cells in forming functional synaptic junctions with neuronal axons, which is largely driven by their electrical activity [38]. Accordingly, OPCs respond to various neurotransmitters released by nearby neurons through expression of variable cell surface receptors, notably the glutamatergic, gamma aminobutyric acid (GABA) receptors and voltage-gated calcium channels (VGCC) (Figure 3) [34,39]. α-Amino-3hydroxy-5-methyl-4-isoxazolepropionic acid (AMPA) type receptors are glutamatergic receptors whose surface expression varies throughout the journey of OPC differentiation into mature OL [39]. Ca^2+^ impermeable AMPA receptor expression is involved in OPC recruitment and proliferation in the adult brain [39,40]. Increased expression of myelin and myelin sheath invagination is found to be N-methyl-D-aspartate (NMDA)-dependent, another glutamatergic receptor highly implicated in OPC differentiation at later stages [41]. As for GABA receptors, their involvement in remyelination and OPC differentiation was noted when a decrease in demyelination was observed in pregnant women with MS [39,42]. The following finding was attributed to an increase in GABA receptor activation by the progesterone metabolite ALLO, which is naturally elevated in pregnancy [39]. In addition to neurotransmitters, OPCs also respond to an array of neuromodulators and neuropeptides that modulate intracellular calcium levels. Such extra-synaptic signaling received from the neuronal activity includes ATP, histamine, serotonin, dopamine, substance P, angiotensin II, and bradykinin [34]. Collectively, those receptors on OPCs help induce an activity-dependent intracellular calcium increase, triggering downstream signaling pathways. Intracellular Ca^2+^ plays a crucial role as a secondary messenger in the axo-glial contact, with increases in intracellular calcium levels reflecting neuronal activity [34]. Rises in intracellular calcium activate the transcription factor SOX10 and upregulate the expression of OLIG2 and NKX2.2. In addition, increased calcium levels stabilize the actin cytoskeleton and allow its polymerization by mediating cortactin phosphorylation (Figure 3) [34].

OLIG1 plays a pivotal role in promoting developmental and non-developmental myelination, as experiments with OLIG1 knockout mice showed reduced remyelination post-demyelinating lesions [5,43]. OLIG2 and NKX2.2 simultaneous expression is a prerequisite for OPC differentiation. OLIG2 subsequently induces the expression of SOX10. Both transcription factors induce the expression of myelin basic protein (MBP) [34]. Additionally, SOX10 is known to induce expression of myelin regulatory factor (MYRF), a transcription factor required for myelin formation in the CNS [34]. In a study by Hornig et al., the selective deletion of the SOX10 gene has been shown to lead to hypomyelination in mice [44]. Emerging research has shown that epigenetic mechanisms, such as DNA methylation, are vital regulators of OPC differentiation. Specifically, Moyon et al. found that DNA methylation regulates the timing of cell-cycle exit during the transition of OPCs to mature and myelinating OLs [45]. In addition, it has been observed that promoters ID2 and ID4 become progressively hyper-methylated during OPC differentiation, while CRISPR/dCas9-DNMT3a-mediated methylation of these promoters promoted OPC maturation [46].

Beyond transcriptional and epigenetic control of OPC maturation, the process is tightly regulated by a complex network of inhibitory and activating pathways linking these cells to their microenvironment. Of the pro-myelinating cascades, stands the PI3K/Akt/mTOR pathway, with its downstream effectors being the protein kinases mTORC1 and mTORC2. While mTORC1 is essential in cytoskeleton reorganization for myelin formation and late-stage OPC maturation, mTORC2 has a more regional but detrimental contribution in early-stage OL formation [47]. On the other hand, the mitogen-activated protein kinase (MAPK) pathway, triggered by a wide array of extracellular signals such as growth factors (FGF-2, PDGF, IGF-I), neurotrophins, neurotransmitters (serotonin), and cytokines (TNF, TGF-β) [48], is particularly implicated in OPC proliferation. Once any of these ligands bind the cell membrane receptors, a three-tiered phosphorylation is triggered in the Ras/Raf/MEK/ERK 1/2 signaling cascade, activating more than 600 cytosolic substrates along with transcription factors of genes involved in cell survival, proliferation, and differentiation [48].

As for inhibitory pathways, the Wingless and integration (Wnt) is a family of ligands expressed upon spinal injury. Of particular interest to the remyelination process is the canonical Wnt/β-catenin pathway. It is an extensively studied signaling cascade that activates transcription factor-like T-cell factor (TCF) and lymphoid enhancer-binding protein (LEF) [49]. Target genes of the pathway include ID2, ID4 and SOX2. ID2 upregulation through Wnt3a increases OPC proliferation, but the activation of the cascade inhibits the differentiation of the OPCs into mature OLs [49]. Nevertheless, contradictory data show that the Wnt/β-catenin signaling cascade could have a pro-myelination feature [49]. In fact, the addition of Wnt1 or Wnt3a in OL-rich culture stimulated the expression of the PLP gene, encoding the primary myelin protein PLP. However, the same was not observed in the OPC culture [49,50]. Therefore, the Wnt/β-catenin has both a positive and a negative correlation with the process of OPC differentiation, depending on the stage at which it is activated, the extent of activation, the downstream activated genes, and the interaction with its surrounding environment.

Another highly regulated pathway with a multifaceted role in remyelination is the NOTCH signaling cascade [23,51]. NOTCH receptors are activated by the ligands Jagged1–2 and Delta-like 1–3, leading to the transcription of target genes primarily associated with the inhibition of differentiation, such as *MYC* and *HES* [23]. Nonetheless, NOTCH signaling exhibits a paradoxical role by promoting OPC proliferation under inflammatory conditions mediated by cytokines such as TGF-β and IL-17, highlighting its temporally regulated function in pathological contexts [52]. Finally, LINGO-1 is a leucine-rich repeat and Ig domain-containing, NOGO receptor-interacting protein, whose expression is increased in several CNS pathologies [53]. The downstream effector of the receptor complex is the RhoA-ROCK (Rho-associated coiled coil containing protein kinase) pathway, whose activation prevents polymerization of actin/tubulin filaments and formation of oligodendrocyte processes [54]. Collectively, these intracellular signaling cascades govern the fate of OPCs in a spatio-temporal fashion by responding to extracellular cues, such as chemokines and cytokines secreted by the activated microglia, astrocytes, and inflammatory cells.

4: Myelin deposition and axon ensheathment: Upon OPC maturation, cytoskeletal remodeling drives the transformation of OPC processes from simple membranous protrusions into multilayered extensions, which continue to elongate as they wrap denuded axons [55]. This process is mediated by the sequential assembly and contraction of actin filaments, followed by actin disassembly promoted by MBP, thereby allowing myelin to glide along the axon [56]. These mechanisms likely account for the variability observed in myelin thickness and internode length, as myelination is locally regulated by individual OPC extensions.

## 3. The Functional Significance of Remyelination

**Figure 4:**
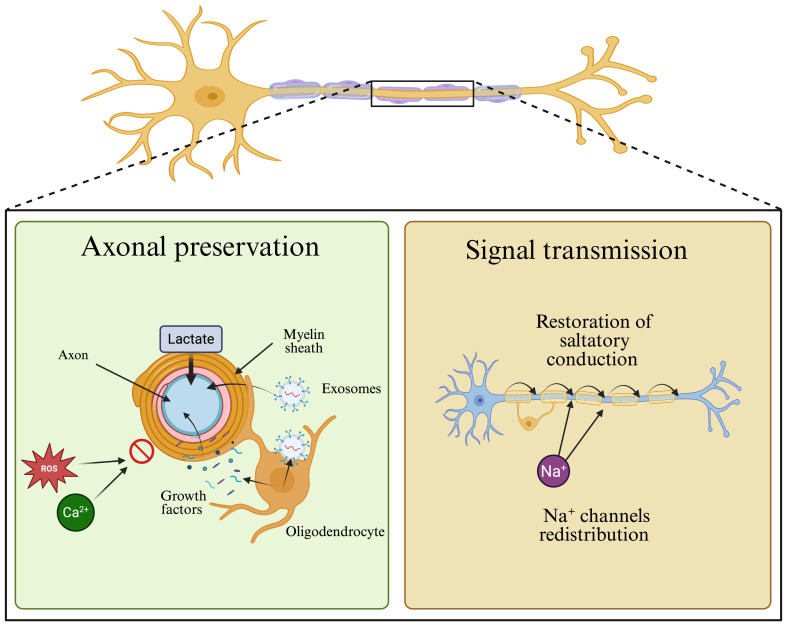
Remyelination’s role in preserving axonal integrity. Axonal preservation (Left): An Oligodendrocyte provides trophic support to neurons through shuttling of metabolites such as lactate or pyruvate. Extracellular vesicles (exosomes) carry lipids, proteins, and RNA supporting myelination and axonal health. Similarly, growth factors secreted by oligodendrocytes, such as IGF-1, maintain axonal growth. Additionally, myelin sheath offers axonal protection by shielding the neuron from reactive oxygen species (ROS) and calcium (Ca^2+^), causing micro-abrasions in its membrane and subsequent axonal degeneration. Signal transmission (Right): remyelination restores faster signal transmission through saltatory conduction and rearrangement of sodium Na^+^ channels, assuring axonal conduction and survival.

Remyelination is an evolutionarily conserved process and constitutes one of the most efficient intrinsic repair mechanisms in the human CNS [57]. The significance of remyelination lies in two key functions: the restoration of axonal conduction and the preservation of axonal integrity [58]. Signal transmission through demyelinated lesions is slowed and may even be blocked due to the absence of saltatory conduction [59,60]. Remyelination reestablishes the clustered nodal distribution of sodium channels, thereby restoring saltatory conduction, a faster and more energy-efficient signal transmission (Figure 4) [59,61]. Nevertheless, the structural properties of newly formed myelin differ from those of developmental myelination. Remyelinated sheaths are thinner relative to axon diameter, a feature commonly described as a higher g-ratio (defined as the ratio of axonal diameter to the total fiber diameter) (Figure 1B–E) [62,63]. In addition, internodes in remyelinated axons are shorter [64], whereas nodal length is increased [65], resulting in an overall reduction in myelin coverage [66]. Nevertheless, these structural differences in the newly formed myelin sheath, compared to developmental myelin, do not affect its neuroprotective role. Mei et al. have demonstrated that an accelerated remyelination in the context of EAE supports axonal integrity and alleviates the severity of EAE clinical scores [67]. So how exactly does remyelination offer axonal protection? Firstly, OLs detect neuronal activity through increases in extracellular potassium levels, resulting from axonal propagation of action potentials [59,68]. This provokes an OL-axon metabolic coupling. OLs provide axons with glycolytic aerobic metabolites, notably lactate, through the myelinic monocarboxylate transporter 1 (MCT1) (Figure 4) [68,69,70]. In fact, heterozygous MCT1-null mice showed widespread axonopathy, despite normal myelination. This highlights the role of shuttled glycolytic end-products like pyruvate and lactate in axonal health maintenance [71,72]. On the other hand, when in a low-glucose environment, OLs fuel enough ATP from fatty acid β-oxidation to prevent axonal loss upon complete depletion of stored glycogen [73].

Another way by which OLs may support axonal survival is through extracellular vesicle (EV) signaling (Figure 4). Exosomes from OLs are excreted in physiological and pathological states and carry proteins involved in myelination and cytoskeleton support, as well as lipids and RNA [71]. Likewise, axons depend on OLs for the secretion of growth factors such as IGF-1 and glial cell-derived neurotrophic factor (GDNF) (Figure 4) [59,71]. Importantly, OLs also protect denuded axons from the detrimental effects of the inflammatory microenvironment observed in various pathological conditions [59,74]. Axonal loss has been linked to inflammation-induced nanoscale disruptions of the axonal membrane, which ultimately trigger intracellular degenerative pathways. Myelination shields the axonal membrane from such micro-insults, preventing toxic increases in intra-axonal calcium levels and subsequent axonal degeneration (Figure 4) [75].

## 4. Remyelination in Multiple Sclerosis (MS)

Focal inflammatory demyelinating lesions evolve with time starting from the acute active phase in which the core of the lesion exhibits intense inflammatory infiltration primarily by activated macrophages containing myelin debris and lymphocytes and ongoing myelin destruction throughout its area [62,76]. Remyelination is primarily observed in the early stages of an active demyelinating lesion, particularly in a period spanning one to two months following its onset [3,65,77]. During this stage, OPCs are recruited and proceed with remyelination from the periphery to the center of the lesion [78]. Chronic active lesions (smoldering lesions) exhibit a core where demyelination is complete with immune cell hypocellularity and an actively demyelinating rim full of myelin-phagocytosing macrophages [79]. In chronic MS, where 57% of lesions are either acutely active or chronically active, the extent of remyelination is inversely correlated to the degree of inflammation [80]. The reason for that is that active lesions may undergo repetitive cycles of demyelination and remyelination (Figure 1A) [3,65]. In chronic inactive lesions, remyelination is heterogeneous: some may undergo full remyelination and appear as shadow plaques, others may undergo partial remyelination in their periphery, whereas others remain totally demyelinated [65,80,81]. In fact, in chronically demyelinated chronic inactive lesions, OPCs are either absent or mostly concentrated on the borders of the lesions but fail to migrate, differentiate, and remyelinate [78,82,83,84].

The extent of remyelination varies widely among MS patients, depending on many intrinsic factors, including the disease course [60]. Endogenous remyelination is most efficient in relapsing-remitting MS (RRMS), followed by primary progressive MS (PPMS) and least efficient in secondary progressive (SPMS) [80,85]. With respect to the duration of the disease, it appears that myelin repair is more efficient in the early stages of the disease and gradually declines with time [77,86].

Lesion localization may also determine the efficiency of myelin repair. Subcortical lesions exhibit more extensive remyelination in comparison to periventricular ones [65,77], and cerebellar lesions largely fail to remyelinate at all [77]. Similarly, inconsistencies exist between the remyelination capacity in the brain and in the spinal cord, with the brain exhibiting a greater ability to undergo the repair [87,88]. In addition, cortical MS lesions are more frequently remyelinated than white matter lesions, in agreement with the higher density of OPCs in cortical lesions, compared to white matter lesions [78,89,90] and a higher density of reactive astrocytes in the white matter lesions, compared to gray matter lesions [90], as OPCs are susceptible to cues secreted by astrocytes [91]. These differences in remyelination potential depending on lesion localization may also reflect the heterogeneity in origin and characteristics of various OPC subpopulations [91,92,93].

In addition to intra-individual variability in the extent of lesion remyelination, substantial inter-individual differences have also been observed. While some individuals exhibit extensive remyelination across multiple lesions, covering up to 96% of the lesion area, others show only sparse and limited remyelination restricted to the lesion periphery [65,66].

Remyelination is influenced by sex-specific differences that arise, at least in part, from hormonal variation [23], which may explain disparities observed between female and male animal models [94]. Sex-related disparities are more complex in humans. Even though MS is more common in females, men are more prone to developing progressive forms of MS, which are associated with reduced regenerative capacity [95]. Nonetheless, despite substantial inter- and intra-individual variability in remyelination potential, the process remains limited [3,65,66,78], and is largely insufficient to halt disease progression in patients with MS. This raises the question of which patient-specific and disease-specific factors impede successful remyelination.

## 5. Why Does Remyelination Fail in MS?

The failure of remyelination in MS results from a myriad of factors overlapping and interfering with the regenerative cascade starting from OPC activation, recruitment/migration, differentiation and myelin deposition [12]. As numerous studies have demonstrated the presence of OPCs in chronically demyelinated MS plaques, OPC exhaustion is not the main cause for remyelination failure [82,84]. In chronic active MS plaques exhibiting either marginal or no remyelination at all, the number of OPCs was found to be decreased compared to healthy controls. Molecules with OPC chemorepellent properties expressed in chronic demyelinating lesions, such as Sema 3A or netrin-1, may be at least partly responsible for the lower OPC densities in chronically demyelinated plaques [37,96,97].

Denuded axons have been shown to express factors inhibiting remyelination, such as LINGO-1 [53,98] or polysialic acid, an inhibitor of developmental myelination also found to be expressed in chronic inactive MS lesions [99,100,101]. Axons in chronic inactive lesions may also stall their own myelin repair by expressing galectin-4, a factor shown to inhibit OPC differentiation [102,103]. Furthermore, evidence suggests OPC differentiation strongly depends on the electrical activity of the axons and suggests that denuded axons that remain chronically electrically inactive may not be amenable to remyelination [104,105].

Immune cells present in chronic inactive lesions may also play a role in preventing remyelination of denuded axons. The M1 microglial phenotype is known to inhibit remyelination, whereas M2 phenotype microglia have been shown to promote OL differentiation and remyelination by secreting mediators such as TNF- α, activin A, or galectin-3 [106,107,108]. In addition, lymphocytes derived from MS patients have been found to inhibit remyelination in experimental models through the secretion of CCL19 [109]. Furthermore, the remyelination inhibitor galectin-4 is also known to be expressed by immune cells [102,103].

As dampening the immune response using corticosteroids has been a standard clinical practice, the impact of the medication on remyelination has been questioned in various experiments. Despite the improvement of symptoms upon short-term high-dose corticosteroid treatment, animal models showed delayed remyelination upon early injection of steroidal treatment [110]. The block in OPC maturation was observed in vitro as well, as pro-maturation factors such as IGF-1 secreted by astrocytes were markedly reduced, while proliferation growth factors increased [111]. Thus, corticosteroid treatment may be double-edged, alleviating inflammatory symptoms while hindering remyelination and OPC differentiation.

Recent evidence suggests that MS is a diffuse disease characterized by a global shift in the functional states of oligodendroglia, rather than just focal demyelination. A study by Jäkel et al. investigates human oligodendrocyte heterogeneity and its role in MS pathology using single-nucleus RNA sequencing (snRNA-seq) on post-mortem brain tissue. By analyzing white matter from healthy controls and various MS lesion types, including active, chronic, and remyelinated lesions, the researchers identified seven distinct OL subclusters, each defined by unique molecular markers. A central finding was that MS significantly alters this cellular landscape. OPCs at an “intermediate” state (Oligo6) connecting precursors to mature cells are markedly reduced in both MS lesions and normal-appearing white matter (NAWM). Furthermore, mature OL populations are skewed; the “Oligo1” subcluster is depleted, while immune-like oligodendroglia (ImOLG) and other subclusters like “Oligo5” are enriched in MS tissue. This discovery of altered heterogeneity provides new insights into disease progression and offers potential targets for regenerative therapies aimed at restoring healthy oligodendrocyte function [112]. Finally, the pattern of chronically demyelinated MS lesions accumulating with increasing age suggests that the failure of myelin repair may also be affected by the biological processes that underlie tissue aging.

## 6. Remyelination and Aging

Aging has been shown to decrease the capacity for myelin repair in several animal models of demyelination [113,114,115,116,117]. Similarly, in MS, the dominant demyelinating lesion type encountered in older patients is that of the chronic inactive lesion, which is either devoid of OPCs or contains OPCs that fail to differentiate and remyelinate. This suggests that the biological processes that drive aging may be a limiting factor for remyelination in advanced stages of multiple sclerosis [83,84].

In a study of remyelination in aged rats, OPCs became unresponsive to pro-differentiation signals. Impaired remyelination capacity was associated with aged rats’ OPCs exhibiting features of cellular senescence with increased levels of DNA damage, mitochondrial dysfunction, CDKN2a and p38MAPK mRNA upregulation [118]. Interestingly, fasting or treatment with metformin has been shown to reverse these changes and restore the regenerative capacity of aged OPCs. It restored their responsiveness to pro-differentiation signals, improving remyelination in aged animals following focal demyelination [118]. This age-related unresponsiveness of OPCs to differentiation signals and the reduction in their population appear to be particularly pronounced in a subpopulation of OPC-expressing acetyl-CoA synthetase 2. Supplementation with acetate (the substrate of acetyl-CoA synthetase 2) appears to preserve the number of OPCs, to promote remyelination after injury, and to improve cognitive function in aged mice [119].

Evidence suggests that microglial aging may also be responsible for less efficient remyelination with age. Microglial and macrophage-mediated clearance of myelin debris, which is a rate-limiting step for remyelination, becomes slower with advancing age [106,117,120]. Nevertheless, microglial cells may also contribute to remyelination by promoting the recruitment and proliferation of OPCs [24]. A recent study using single-cell RNA sequencing to study the response of microglia during remyelination in the lysolecithin mouse model observed a delay in the appearance of the expected microglial states with age, in concordance with delayed remyelination [121]. In addition, senescence-associated pro-inflammatory mediators such as CCL11/Eotaxin-1 secreted by aged microglia have been shown to limit remyelination by inhibiting oligodendrocyte maturation [122].

Cellular senescence has been recognized as a key biological process underlying normal aging, and evidence suggests that senescent cells accumulate with time [123,124]. The senescent phenotype is typically associated with several metabolic and functional changes, including stable cell cycle arrest, the expression of a pro-inflammatory senescence-associated secretory phenotype (SASP), and the accumulation of dysfunctional mitochondria [125,126,127,128]. Using markers of cellular senescence, we recently found evidence of a significantly greater load of senescent cells in autopsy material from MS patients than in age-matched controls. Senescent olig+ oligodendrocyte/oligodendrocyte progenitors in cortical demyelinating MS lesions ranged from 20.2 to 33.1% of all senescent cells, and senescent microglia from 8.8 to 22.6% of all senescent cells, depending on the colocalization markers used [129]. The cell-cycle arrest and extensive gene expression changes that occur in senescent cells—reflecting a severe loss or alteration of normal physiological function—may contribute to age-related changes in OPCs and microglia, thereby reducing the efficiency of remyelination with advancing age [130].

Neural progenitor cells that normally promote OPC differentiation have been shown in demyelinated lesions from PPMS patients to acquire a senescent phenotype and secrete high mobility group box protein 1 (HMGB1), which drives OPCs into a senescent state. Blockade of the HMGB1 senescence mediator promoted OPC differentiation in vitro, indicating that accelerated aging of neural progenitor cells from demyelinating lesions may also adversely affect spontaneous remyelination [131]. The role of cellular senescence in age-related failure of remyelination is reviewed by Koutsoudaki et al. [132] and Maupin & Adams [133].

## 7. What Is the Evidence That Remyelination May Be Beneficial?

Multiple electrophysiological studies in animal models have demonstrated a clear link between the restoration of myelin in the CNS and the recovery of axonal conduction, consistently associated with the alleviation of neurological symptoms [134,135]. Evidence from several transgenic mouse models further supports the protective role of myelin, as the deletion of individual myelin-related genes—such as CNP and PLP—results in secondary axonal degeneration accompanied by functional impairments [136,137].

This neuroprotective effect is mediated through multiple mechanisms, including restoration of nodal architecture, physical insulation of demyelinated axons from a hostile extracellular milieu, and re-establishment of metabolic support [138]. For instance, Nav1.6 voltage-gated sodium channels, which generate persistent sodium currents [139] become diffusely distributed along demyelinated axons, leading to hyperexcitability [140] and increased vulnerability to injury [141]. Remyelination has been shown to normalize sodium channel clustering as mentioned earlier [142,143]. In addition, sustained electrical activity in the setting of ionic imbalance may drive elevated metabolic demand, consistent with the increased mitochondrial content observed in denuded axons [144]. Interestingly, remyelinated axons exhibit even higher mitochondrial density in both MS tissue and experimental demyelination models [145], likely reflecting a compensatory response to their heightened energy requirements.

Pathological studies in experimental models and MS autopsy tissues support the neuroprotective role of remyelination, as evidence has demonstrated lower levels of acute axonal damage and loss in remyelinated lesions compared to non-remyelinated ones [67,146,147,148]. In addition, thinner caliber myelinated fibers, which are ensheathed with a thinner myelin sheath, are more susceptible to degeneration under conditions of inflammatory demyelination, compared to thicker ones. This indicates that the thicker the myelin sheath, the greater the neuroprotection from the detrimental effects of the inflammatory milieu [28,149]. Furthermore, neuropathological evidence suggests an inverse correlation between the extent of remyelination in the spinal cord of progressive MS patients and their level of disability [85].

A longitudinal in vivo PET scan–MRI combined-based study quantifying myelin destruction and repair in patients with active relapsing–remitting MS and healthy controls found a significant association between the patients’ remyelination potential and their level of disability as measured with the Expanded Disability Status Scale (EDSS) score [150]. Another MRI-based study using the newly described x-separation method for quantifying remyelination found that in young MS patients, changes in myelin content indicative of remyelination correlated with reductions in EDSS scores [151].

## 8. Is Remyelination Clinically Meaningful? Insights from Clinical Trials of Remyelinating Agents and Future Directives

The concept of remyelination in MS represents a pivotal shift in therapeutic strategy, moving from suppressing the pathogenic immune response and containing inflammation with disease-modifying treatments (DMTs) to the active restoration of the myelin sheath. Consequently, enhancing endogenous remyelination is widely regarded as the most tractable strategy to delay, prevent, or potentially reverse disability in MS.

Insights from the first generation of clinical trials on remyelination have provided a “proof-of-concept” that biological repair is achievable in the adult human brain, yet they have also highlighted the difficulty of translating these into gains in functional clinical outcomes. A systematic review of 25 studies involving 17 different interventions revealed that several agents—most notably clemastine fumarate, opicinumab, and rHIgM22—exhibited distinct remyelination potential [152].

Clemastine fumarate, a type 1 muscarinic and type 1 histamine receptor antagonist, promoted OPC differentiation and remyelination in preclinical models [153,154,155,156]. The ReBUILD trial of clemastine fumarate was a landmark success, demonstrating a statistically significant reduction in visual evoked potential (VEP) latency. While VEP latency improved, the clinical meaningfulness of this change was subtle, as it did not immediately translate into dramatic shifts in gross disability scales such as EDSS [157]. Moreover, the TRAP-MS trial in advanced progressive MS raised safety concerns and reported adverse effects, highlighting population-specific considerations for efficacy and tolerability [158]. TRAP-MS patients, on average, also had a much longer disease duration (mean duration 22.3 years) and had entered the progressive phase of disease. It was postulated that clemastine accelerates MS-related disability accumulation in this population by enhancing pyroptosis [158]. Nevertheless, it is also plausible that older patients with progressive MS may be more susceptible to antihistamine side effects (e.g., fatigue and confusion) that could skew performance-based measures. Ongoing trials (ReINFORCE, RESTORE, ReVIVE) continue across MS populations, with mixed signals regarding generalizability [159].

Similarly, opicinumab, a monoclonal antibody against LINGO-1, was investigated across several trials. RENEW showed potential VEP improvements in acute optic neuritis but no definitive intention-to-treat (ITT) efficacy; follow-up analyses suggested some electrophysiologic benefits but no robust effects on disability outcomes [160]. However, the SYNERGY and AFFINITY trials in RRMS/SPMS did not demonstrate clear dose–response or clinical efficacy [161,162]. In particular, the SYNERGY trial failed to meet its primary endpoint of dose-linear improvement. However, post hoc analyses suggested that younger patients with shorter disease durations might still derive benefit. Similarly, in the AFFINITY trial, the primary endpoint (Overall Disability Response Score-ODRS over 72 weeks) was not met, although numerical trends favored opicinumab. Interestingly and in contrast to the findings of the SYNERGY study, the AFFINITY study showed that participants of older age, higher EDSS and longer disease durations may benefit more from opicinumab infusions, suggesting that severe tissue damage at baseline may limit the potential for remyelination [162]. The sponsor subsequently discontinued drug development. Several factors might have contributed to the failure of opicinumab, including first the failure to capture the therapeutic window—in RENEW, most macular thinning had already occurred before enrolment, which was within 4 weeks of symptom onset; the heterogeneous patient selection—SYNERGY included both relapsing and progressive multiple sclerosis populations, potentially diluting treatment effects given differences in endogenous remyelination capacity and the ambitious outcome selection—the trial was designed to detect disability improvement rather than slowing of progression over 18 months [159]. Despite these failures, the opicinumab studies have provided the largest dataset from remyelination trials to date. Their results inform key design considerations for future remyelination trials, suggesting that remyelination may be most beneficial in patients receiving disease-modifying treatment with dimethyl fumarate, with a mid-range level of disability, and that more robust, remyelination-specific imaging biomarkers—based on advanced MRI techniques—may be required, along with higher doses and longer treatment durations.

Bexarotene, a nonselective retinoid X receptor γ (RXRγ) agonist approved by the US Food and Drug Administration (FDA) for the treatment of cutaneous T cell lymphoma, was explored in the CCMR One trial. The primary endpoint (patient-level change in the magnetization transfer ratio (MTR) within lesions exhibiting baseline MTR values less than the within-patient median) was not met, but modest MRI and electrophysiologic signals suggested some remyelination. Bexarotene significantly increased lesional MTR in gray matter, but not in white matter lesions and improved P100 peak time in chronically demyelinated eyes by 4.06 milliseconds. These results were most pronounced in younger patients, suggesting age and region-specific responses [163]. However, significant toxicity (all patients experienced at least one adverse event, most commonly central hypothyroidism, hypertriglyceridemia, headache, and rash curtailed further testing. According to this trial, gray matter lesions may offer the most sensitive target for detecting treatment-related changes in lesional MTR, though it is unclear whether this effect may hold true for drugs with other mechanisms of action.

GSK239512, an H3 receptor inverse agonist, was studied in a phase 2 single-blind trial in RRMS patients as an add-on to interferon-β1a or glatiramer acetate. The co-primary endpoints were changed in MTR in gadolinium-enhancing and non-enhancing lesions with a reduction in MTR greater than the 99th percentile of normal white matter MTR variation (termed delta-MTR lesions). A modest statistically significant effect was observed in Gd+ lesions but not in delta-MTR lesions and in secondary clinical endpoints. Trial power reduced statistical power due to a high drop-out rate in the active treatment group and an unexpectedly low rate of new MRI lesion formation among participants, along with off-target effects, may have influenced outcomes [164].

GNbAC1 (temelimab) is a monoclonal antibody targeting the HERV-W Env protein, a proposed contributor to MS pathogenesis. It has been hypothesized to reduce HERV-W–mediated inflammation and to potentially promote myelin repair. In the CHANGE-MS phase IIb trial, multiple doses of GNbAC1 were evaluated in RRMS. At 48 weeks, patients continuously treated with high-dose GNbAC1 showed MRI evidence of reduced brain atrophy, reduced conversion to T1 black holes and MTR evidence of preserved myelin integrity [165]. Despite the encouraging results of this study, no direct evidence of GNbAC1 promoting remyelination has been provided to date.

Domperidone, a D2/D3 dopamine antagonist, which increases prolactin levels, was investigated in RRMS and SPMS. In a randomized, controlled pilot trial with domperidone as an adjunct therapy in 17 RRMS patients on DMT, MRI texture and fractional anisotropy measures indicated earlier lesion repair in the domperidone group, compared to the control group at 16 weeks of treatment probably reflecting a prolactin-mediated remyelinating effect [166]. Nevertheless, a larger futility trial in SPMS did not show benefit. Recruitment and lesion identification posed challenges and it did not substantiate remyelination efficacy [167].

Phenytoin demonstrated a significant neuroprotective effect on macular ganglion cell-inner plexiform layer (mGC-IPL) thickness at 6 months in unilateral acute optic neuritis, compared with placebo. This is consistent with the attenuation of retrograde degeneration via sodium-channel blockade. Preserved mGC-IPL thickness correlated with favorable electrophysiologic function (VEP latency and amplitude), indicating functional preservation alongside structural protection. The beneficial effect of phenytoin on mGC-IPL appeared more pronounced at worse baseline visual acuities, suggesting clinical relevance for patients with greater initial impairment. The study provided Class II evidence supporting phenytoin’s association with preserved mGC-IPL in acute optic neuritis [168].

Several agents continue in phase II or III, including BIIB061, Bazedoxifene, Ifenprodil, PIPE-307, testosterone and metformin, focusing on OPC differentiation, energy metabolism, or hormonal pathways, with varying degrees of preclinical support and clinical progress. Testosterone and synthetic analogs acting on the brain androgen receptor have been shown to efficiently promote remyelination and suppress neuroinflammation in the cuprizone model of demyelination in mice [169] and is now being tested in MS patients [170]. The Octopus Trial (Multi-Arm Multi-Stage) is currently testing Metformin and Lipoic Acid in progressive MS patients. As of February 2026, it has enrolled over 700 participants, making it one of the largest progressive MS trials ever conducted. On the other hand, the Belgian MACSiMiSE-BRAIN study focuses specifically on examining the effect of metformin as a standalone add-on, in non-active progressive MS patients, on walking speed, as examined with the Timed 25-Foot Walk (primary end point) after a 96-week treatment period [171].

More recently, the preliminary results of the CCMR-Two clinical trial, which investigated the effects of a combination of clemastine and metformin on remyelination in MS, were presented at the ECTRIMS 2025 congress. The trial involved 70 participants with RRMS who were already stable on DMT. The combination improved VEP latency (primary endpoint), but there were no significant improvements in visual function or overall EDSS scores during the 6-month study period. The combination was generally well-tolerated, though side effects were common, particularly fatigue associated with clemastine and gastrointestinal issues associated with metformin. Nonetheless, these data are still preliminary and have not yet been subject to full publication and review [172].

All in all, completed clinical trials have not demonstrated to date a robust, clinically meaningful benefit of remyelination (Table 1). However, they have substantially strengthened the evidence that promoting remyelination is feasible in clinical practice, supported by convergent electrophysiological and neuroimaging markers. Importantly, these studies provide encouraging preliminary signals that sustain remyelination as a viable neuroprotective strategy and have generated critical insights to inform the design of next-generation trials.

Advancing the field will likely require a more refined approach to patient selection, biomarker choice, and trial duration. Central to identifying the optimal target population is the recognition that successful remyelination depends on the presence of surviving axons. Histopathological studies demonstrate that axonal damage and loss are greatest during the early acute phase of demyelinating lesion formation, followed by a slower but sustained phase of axonal degeneration during chronic lesion evolution [147,148,173]. It is therefore unlikely that remyelination therapies can salvage axons lost during this initial inflammatory stage.

Thus, the timing of intervention appears critical for remyelination to translate into a clinically meaningful benefit. The therapeutic window for preventing long-term disability is likely narrow, with optimal outcomes achieved when treatment is initiated early in the disease course, at a stage when axons remain viable, electrically active, and receptive to myelin repair [104,105,159]. In contrast, during the progressive stages of MS, tissue-restricted inflammation and compartmentalized pathology create a metabolically hostile environment for both axons and remyelinating cells. Concomitant glial metabolic reprogramming toward glycolysis may further deprive neurons of essential energy substrates, exacerbating neuronal stress and impairing OPC differentiation [166]. Consequently, patients with advanced progressive MS are unlikely to derive significant benefit from remyelinating therapies and should not be the focus of early-stage clinical trials.

To date, the majority of remyelination trials have been conducted in patients with optic neuritis (Table 1). Optic nerve demyelination provides a particularly advantageous experimental system, owing to its well-defined structure–function relationship. However, remyelination effects observed in the optic nerve may not be directly generalizable to other regions of the CNS, as accumulating evidence suggests that endogenous remyelination capacity may vary substantially according to lesion location [78,90]. Trials in acute optic neuritis represent a valuable Phase II proof-of-concept model for establishing the remyelinating potential of candidate therapies. Nevertheless, greater external validity may be achieved by extending these approaches to younger patients with relapsing MS, shorter disease duration with minimal or no radiological activity under effective immunomodulatory treatment. In this population, the addition of a remyelinating agent—should it prove efficacious—would be more likely to yield clinically meaningful and generalizable benefits.

Finally, mounting evidence indicates that MS is a far more diffuse disease than suggested by focal demyelinating lesions alone, with profound alterations in oligodendrocyte heterogeneity evident even within NAWM [112]. These transcriptomic and functional shifts suggest that remyelination failure in MS does not simply reflect insufficient oligodendrocyte numbers. It is rather a systemic reprogramming of the oligodendroglial lineage, in which cells may adopt immune-responsive or stress-associated states at the expense of efficient remyelination.

In response to these intrinsic and extrinsic barriers, clinical trials are increasingly exploring combination therapeutic strategies that integrate remyelination with neuroprotection and immunomodulation. For example, targeting regulated cell-death pathways such as necroptosis or ferroptosis—both implicated in axonal and oligodendroglial degeneration in people with MS [174]—may preserve axonal integrity long enough to provide a viable substrate for remyelinating agents to exert meaningful effects. Similarly, given the strong association between aging and impaired spontaneous myelin repair, interventions aimed at blocking or reversing biological processes of aging, including cellular senescence, may enhance the efficacy of remyelinating therapies [133,175]. Targeting mechanisms of cellular aging have yielded promising results in both preclinical models and early clinical trials [118,172].

**Table 1:** Clinical trials of remyelinating agents in multiple sclerosis.

Medi Cation/ Trial Name	MoA	Population	Design	Mean Age (Years ± SD)	Trial Duration (Weeks)	Phase of Trial	Primary End-Point	Status	Outcome	References
Bazedoxifene (ReWRAP) (NCT04002934)	Selective estrogen receptor modulator	Post-menopausal females with RRMS or chronic ON on immunomodulatory therapy	Randomized, Placebo-controlled, Double-blind, Delayed start	52.5 ± 6.25	24	Phase 2	MRI-based Myelin Water Fraction in the corpus callosum compared to placebo	Completed	The primary endpoint was not met	[176]
GSK239512 (NCT01772199)	BBB crossing, H3 receptor antagonist and inverse agonist	RRMS on a stable dose of interferon-beta-1a IM or glatiramer acetate SC	Randomized, parallel group, placebo-controlled	34 ± 8	48	Phase 2	Mean change in lesion GdE and lesion Delta MTR differences	Completed	The primary endpoint was met	[164]
Opicinumab (RENEW)	Human anti-LINGO-1 monoclonal antibody	Patients with the first acute ON	Add-on/parallel, Randomized, Placebo controlled, Double blind	32.1 ± 8	24	Phase 2	Change in Full-Field VEPs by week 24.	Completed	Primary endpoint not met	[162]
Bexarotene (CCMR ONE) (EudraCT 2014-003145-99)	Retinoid X Receptor (RXR) agonist	Adult RRMS patients on dimethyl-fumarate	Two-center, randomized, Placebo-controlled, Double blind	34 ± 8	24	Phase 2a	AEs and withdrawals attributable to bexarotene & change in mean lesional MTR between baseline and month 6	Completed	Primary endpoints not met: poor tolerability and no difference in MTR	[177]
Opicinumab (AFFINITY)	Human anti-LINGO-1 monoclonal antibody	Adults RRMS patients on DMT and with evidence of recent relapse	Multicentre, Randomized, Double-Blind, Placebo-Controlled, add-on therapy to standard DMTs	38.6 ± 9.2	72	Phase 2	Overall Disability Response Score (ODRS)	Terminated	Primary endpoint not met	[178]
Domperidone (NCT02493049)	Peripheral D2 receptor antagonist	Adult RRMS patients with ≥1 GdE lesion and n DMT therapy	Randomized controlled, open label	40.2 ± 4.7 *	144	Phase 2	Recruitment of 24 participants within 36 months with a 79% completion rate and MRI outcomes of lesion repair using texture analysis, MTR and DTI	Completed	Primary endpoint not met	[166]
Opicinumab (SYNERGY)	Human anti-LINGO-1 monoclonal antibody	Adult RRMS and relapsing SPMS on interferon beta-1a IM	Multicentric, randomized, double blind, placebo-controlled, add-on, dose-ranging	39.5 ± 9.29	72	Phase 2	CDI over 72 weeks based on EDSS, T25FW, 9-Hole Peg Test, and PASAT-3	Completed	Primary end point not met: no significant dose-linear improvement in disability compared with placebo	[161]
Clemastine fumarate (ReBUILD)	Competitive H1 receptor and M1 antagonist	relapsing multiple sclerosis with chronic ON on stable immunomodulatory therapy	Randomized, Double-Blind, Placebo-Controlled, Crossover	40.1 ± 10.2	20	Phase 2	Reducing P100 latencies on full field transient pattern reversal VEPs	Completed	Primary endpoint met: significant reduction in VEP latency	[157]
Clemastine fumarate (ReCOVER)	Competitive H1 receptor and M1 antagonist	Patient with Acute ON	Randomized, Double-Blind, Parallel-Group, Placebo-Controlled	N/A	36	Phase 2	Change in P100 latency on full-field VEP and Change in low contrast visual acuity	Recruiting	Ongoing	[179]
Transorbital electrical stimulation (ONSTIM)	Non-invasive stimulation delivers weak electrical currents to the eye, stimulating retinal neurons	Adult RRMS patients with Acute ON	Randomized, Parallel assignment, Double-masking	N/A	24	N/A	Modification of the latency of P100 wave on VEPs after 24 weeks of treatment with electrical or sham stimulation	Recruiting (last known update 2019)	N/A	[180]
Transorbital electrical stimulation (NCT03862313) (ACSON)	Non-invasive stimulation delivers weak electrical currents to the eye, stimulating retinal neurons	Adult with first-ever ON with or without established multiple sclerosis	Randomized, single-blind, parallel, sham-controlled pilot Study	N/A	16	N/A	Low-contrast visual acuity, macular GCL-IPL thickness on OCT and total number of AEs	Terminated due to insufficient recruitment	N/A	[181]
Nanocrystalline gold (VISIONARY-MS) (NCT03536559)	Catalytic conversion of NADH to NAD+	Adults with RRMS with maximum best corrected BC-HCVA or Chronic ON	Randomized, Double-blind, Parallel-group, Placebo-controlled study	36.5 ± 9.25	48	Phase 2	Change in BC-LCLA	Terminated due to COVID-19-related enrolment issues	N/A	[182]
Testosterone (TOTEM-RRMS) (NCT03910738)	Androgen receptor agonist	Male patients with RRMS on ≥1 DMT therapy in the past and biological hypogonadism	Multicentre, Randomized, Parallel-group, Double-blind, Placebo-controlled	N/A	66	Phase 2	Thalamic atrophy <0.5% and modification in transverse diffusivity of lesions <0.5% per year on MRI	Recruiting	Ongoing	[170]
combination of metformin and clemastine (CCMR TWO) NCT05131828	Metformin: inhibitor of mitochondrial respiratory chain complex I and an AMPK activator. Clemastine: a competitive H1 receptor and M1 antagonist	RR-MS patients with chronic stable ON in at least one eye	Randomized, placebo-controlled, double-blind trial	N/A	24	Phase 2	Change in the P100 latency of the full-field VEP between baseline and week 26	Completed	Primary end-point met	[172]

Abbreviations: AEs: adverse events; AMPK: AMP kinase; BBB: blood–brain barrier; BC-HCVA = Best Corrected High Contrast Visual Acuity; BC-LCLA = Best-Corrected Low-Contrast Letter Acuity; CDI = Confirmed Disability Improvement; DMT = Disease Modifying Therapy; DTI: diffusion tensor imaging; EDSS = Expanded Disability Status Scale; GdE = Gadolinium enhanced; H1/3 = Histamine 1 or 3 receptor; M1: muscarinic acetylcholine receptor; LINGO-1 = Leucine rich repeat, Ig domain containing; Nogo receptor interactive protein-1; MoA = mechanism of action; MRI = Magnetic Resonance Imaging; MTR = Magnetization Transfer Ratio; N/A: not available; NADH = Nicotinamide Adenine Dinucleotide + Hydrogen; ON = optic neuritis; PASAT-3: Paced serial additions test at 3 secs; T25FW = Timed 25-Foot Walk; VEP = Visual Evoked Potential. * Mean ages and SDs estimated from the reported medians and ranges. Trials that do not assess remyelination as their primary outcome, such as the CHANGE-MS, have been excluded from the table.

## 9. Conclusions

Great progress has been made in elucidating the cellular and molecular interactions that underpin and facilitate myelin repair in demyelinating disease. Our current understanding of the molecular mechanisms driving myelin repair is creating new opportunities to target multiple pathways with the aim of promoting remyelination. Enhancing remyelination may address the core driver of irreversible disability in multiple sclerosis: the loss of axonal integrity and metabolic support.

As the current single-faceted, immunomodulation-based therapeutic strategy has failed to prevent chronic neurodegeneration–driven disability progression, add-on therapies that promote myelin repair may represent a game-changing approach for progressive MS. Although numerous compounds have shown promise in vitro and in experimental models of demyelination, clinical trials of remyelinating agents to date have provided minimal evidence of meaningful clinical benefit. This lack of robust efficacy data likely reflects several factors, including small trial sample sizes, missing the optimal time window when axons are still amenable to remyelination, short follow-up periods, suboptimal combination with only moderately effective immunomodulators, and the absence of standardized endpoints that reliably link biological repair to real-world functional recovery.

We postulate that because myelin repair is impaired by a range of factors that modify the functions of multiple cell types involved in the repair process, effective treatment may require combining agents that act on several complementary remyelination-promoting targets. Such an approach could help restore the diverse functional states of the various cell populations required for endogenous myelin repair. Achieving this could deliver a clinically meaningful neuroprotective effect capable of preventing disability progression and improving the long-term quality of life for individuals with MS.

Finally, we acknowledge that this article is a narrative review and, as such, may be subject to selection bias, particularly given the rapidly evolving nature of the remyelination field and the continuous emergence of new experimental and clinical data. Moreover, many of the mechanistic insights discussed are derived from animal models or in vitro studies, which may not fully recapitulate disease pathogenesis in humans or reliably predict therapeutic efficacy in clinical settings. Therefore, ongoing updates through translational research and systematic reviews remain essential.

###  Search Strategy

A literature search was conducted using Pubmed, Medline, and Google Scholar using the following search terms: “multiple sclerosis”, “remyelination”, “demyelination”, “neurodegeneration”, “oligodendrocyte”, “oligodendrocyte precursor cells”, and “oligodendrocyte progenitor cells”. Emphasis was placed on peer-reviewed original research articles, systematic reviews, and recent narrative reviews in English. Additional publications were obtained through screening of reference lists in selected articles. As for therapeutic trials, we searched Clinicaltrials.gov and the EU Clinical Trial register for recent trials in phase 2 or above, with primary or secondary outcomes being remyelination or functional regain. A screening of the ECTRIMS website was performed to include recent abstracts and proceedings presented at the latest ECTRIMS 2025.

## Data Availability

Not applicable.
